# Variable training but not sleep improves consolidation of motor adaptation

**DOI:** 10.1038/s41598-018-34225-w

**Published:** 2018-10-29

**Authors:** Benjamin Thürer, Frederik D. Weber, Jan Born, Thorsten Stein

**Affiliations:** 1 BioMotion Center, Institute of Sports and Sports Science, Karlsruhe Institute of Technology, 76131 Karlsruhe, Germany; 2 Institute for Medical Psychology and Behavioral Neurobiology, University of Tübingen, 72074 Tübingen, Germany; 3 Donders Institute for Brain, Cognition, and Behaviour, Radboud University Medical Centre, 6525 EN Nijmegen, The Netherlands

## Abstract

How motor memory consolidates still remains elusive. Consolidation of motor skills has been shown to depend on periods of sleep. Conversely, motor adaptation during tasks not dependent on the hippocampus may not depend on sleep. Some research suggests that the training schedule affects the sleep dependency of motor adaptation tasks. Here, we investigated whether sleep differentially affects memory consolidation that depends on the training schedule. Healthy men were trained with their dominant, right hand on a force-field adaptation task and re-tested after an 11-h consolidation period involving overnight sleep (Sleep) or daytime wakefulness (Wake). Retesting included a transfer test of the non-dominant hand. Half of the subjects in each group adapted to different force-field magnitudes during training with low inter-trial force variability (Sleep-Blocked; Wake-Blocked), and the other half were trained with a high-variability schedule (Sleep-Random; Wake-Random). EEG was recorded during task execution and overnight polysomnography. Consolidation was comparable between Wake and Sleep groups, although performance changes over sleep correlated with sleep spindles nesting in slow-wave upstates. Higher training variability improved retest performance, including transfer learning, and these improvements correlated with higher alpha power in contralateral parietal areas. These enhanced consolidation effects might be fostered by feedback rather than feedforward mechanisms.

## Introduction

The influence of post-learning sleep on motor memory consolidation has been frequently investigated^1^. However, the literature shows an inconsistent picture, with some studies supporting (e.g.^2–4^) and others not supporting (e.g.^5,6^) sleep-dependent consolidation of motor memory. Hence, research points towards a complex relationship between specific aspects of motor tasks and sleep^7^ (but also see^8^). A recent qualitative literature review^1^ identified studies that have found that the motor benefits or stabilization due to sleep can be seen in explicit sequence learning tasks (e.g.^9,10^), specific variants of implicit sequence-learning tasks (e.g.^11–13^), and specific visuomotor adaptation tasks (e.g.^14^). However, all these tasks involve hippocampal function to a certain extent. It has been suggested that specific nonhippocampal-mediated tasks, like motor adaptation to dynamic perturbations (e.g., force field adaptation^15^), reflect a motor-memory process that is purely time-dependent, but not sleep-dependent^5,16^. Although motor adaptation studies support this view^17,18^, those results, to the best of our knowledge, have not been confirmed, to date, using a standardized sleep protocol.

Besides sleep-dependent effects on specific aspects of tasks, there could be effects of specific training schedules. Several studies have shown that motor training under highly variable conditions (variable in terms of random changes in movement kinematics or dynamics) enhance posttest and transfer performance, suggesting that, depending on the training schedule, different memory systems are involved (e.g.^19–21^). Though it has been assumed that the benefits of variable training depend on sleep^1^, this assumption is based on a study of imagery training, which showed that variable training, but not constant mental training, on a motor task led to sleep-dependent memory improvements^22^. However, other studies have revealed that the hippocampal dependency of a motor task changes with the training schedule and the amount of training^23–25^.

In this study, we assessed the effects of sleep on motor memory consolidation in a force-field adaptation task. Specifically, we were interested in whether effects of sleep would depend on the variability of the training schedule. For this purpose, subjects either trained with a low inter-trial force variability or a high inter-trial force variability and were retested after periods of sleep or wakefulness with the same hand. Since previous work from our laboratory showed sleep-dependent consolidation effects for contralateral transfer^26^, we also examined transfer performance on the contralateral hand. We recorded EEG correlates during training, intervening sleep, and retesting, with the aim of characterizing the role of online-feedback mechanisms to mediate improvements during movement execution.

## Results

**Figure 1:**
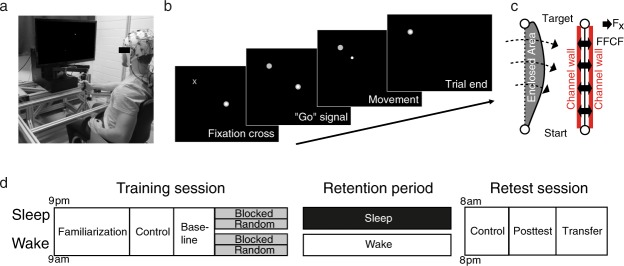
(**a**) The motor adaptation task was performed using a robotic manipulandum (Kinarm End-Point Lab, BKIN Technologies) with a custom made low-friction air-sled system. The robotic manipulandum could induce force fields to perturb participants’ hand movements. During the task, each participant’s EEG was recorded. (**b**) Example of one trial from the highlighting of the fixation cross until the trial ended by reaching the target. (**c**) Sketch of the parameters quantifying motor error (enclosed area, EA) and the adaptation coefficient (force-field compensation). Enclosed area (left) was defined by the area between the trajectory and the straight line between the start point and the target. Arrows indicate the force-field direction. The adaptation coefficient (right) was computed using the subject’s forces (F_x_) directed against virtual channel walls and compared to the ideal force profile to cancel out the perturbation. (**d**) All participant groups had a Training session in which they performed the motor adaptation task with their dominant right hand, including a Familiarization phase, Control tests (subjective sleepiness, mood, and vigilance), Baseline, and the force-field Training (gray blocks). After a Retention period, Retest performance was quantified in a Posttest and an additional Transfer test with the left hand. Groups differed in their training and consolidation periods. The Random groups were trained on the motor adaptation task under highly variable force-field conditions and the Blocked groups were trained under more stable conditions. The Wake groups trained in the morning and were retested in the evening and the Sleep groups were trained in the evening and retested the following morning after a night of sleep.

Similar to a previous study from our laboratory^27^, participants performed a point-to-point reaching task using a robotic manipulandum (Fig. 1a,b). The manipulandum induced a force field that perturbed participants’ reaching movements, and thus, decreased their motor performance. The force-field magnitudes varied when participants either practiced the reaching movements in a blocked (low inter-trial force field variability) or a random (high inter-trial force field variability) training schedule. Motor error was quantified by the enclosed area (EA) of the hand trajectory (Fig. 1c, left) with respect to the straight hand path. Additionally, we quantified motor performance on so-called error clamp (force channel) trials (Fig. 1c, right). During these trials, force-field compensation between the measured and the ideal force profile was computed^28^, mainly reflecting movement predictions, and thus, feedforward mechanisms^29^. Henceforth, the term motor error will refer to EA and the term adaptation coefficient will refer to force-field compensation.

Participants were assigned to four groups that practiced on either a random or a blocked training schedule during the first test session, with half the participants staying awake and the other half going to sleep after the first session (Fig. 1d). The groups were named, accordingly: Wake-Random (WR), Wake-Blocked (WB), Sleep-Random (SR), and Sleep-Blocked (SB). All participants performed the same task during a second test session, including a Posttest with a constant, average force field, and a Transfer test using their left, non-dominant hand. Electroencephalography (EEG) was recorded for all the participants during Training, the Posttest and the Transfer test, and polysomnographic recordings were made for all the Sleep participants.

### Behavioral results

**Figure 2:**
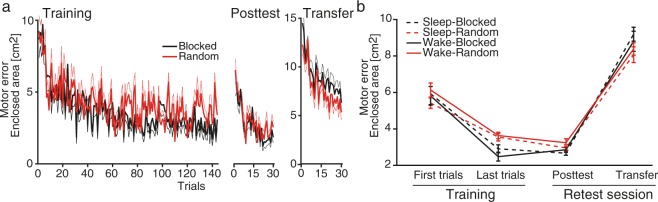
(**a**) Progress of the mean (thick solid lines ± S.E.M, fine dotted lines) motor error (enclosed area, EA) for the Blocked (black) and Random (red) groups during Training (left), Posttest (middle) and Transfer trials (right). (**b**) Mean (±S.E.M) motor error across 30 trials for each group during First and Last Training Trials, Posttest and Transfer.

The main behavioral results are presented in Table 1. The groups adapted to the force fields and decreased their motor error during Training, independent of Sleep/Wake conditions or Blocked/Random training conditions (quantified by enclosed area, EA; Fig. 2a,b). The Blocked groups adapted faster during Training than the Random groups, which was confirmed by FDR corrected *post-hoc t*-tests on the Last Training Trials between the Random and Blocked groups (*t*(46) = 3.96, *p* = 0.002, *d* = 1.144).

**Table 1:** Results of ANOVAs on motor error and adaptation coefficient.

n = 48		motor error			adaptation coefficient		
		*F*	*P*	pη²	*F*	*P*	pη²
Adaptation	time	143.05	**<0.001**	0.77	351.91	**<0.001**	0.89
	time*sleep	1.29	0.262	0.03	0.83	0.369	0.02
	time*practice	4.50	**0.040**	0.09	1.99	0.165	0.04
	time*practice*sleep	0.20	0.667	0.00	0.02	0.894	0.00
	sleep	0.06	0.802	0.00	0.05	0.823	0.00
	practice	1.95	0.169	0.04	0.06	0.811	0.00
	sleep*practice	1.36	0.250	0.03	0.04	0.841	0.00
Consolidation	time	4.30	**0.044**	0.09	69.23	**<0.001**	0.61
	time*sleep	1.68	0.201	0.04	1.23	0.274	0.03
	time*practice	6.95	**0.012**	0.14	0.56	0.459	0.01
	time*practice*sleep	1.44	0.237	0.03	1.93	0.172	0.04
	sleep	0.02	0.896	0.00	0.00	0.965	0.00
	practice	12.70	**<0.001**	0.22	0.57	0.453	0.01
	sleep*practice	0.39	0.537	0.01	1.11	0.287	0.03
Generalization	time	483.56	**<0.001**	0.92	425.43	**<0.001**	0.91
	time*sleep	0.11	0.742	0.00	1.37	0.249	0.03
	time*practice	16.57	**<0.001**	0.27	0.41	0.524	0.01
	time*practice*sleep	0.17	0.680	0.00	0.00	0.979	0.00
	sleep	0.71	0.403	0.02	0.09	0.771	0.00
	practice	0.09	0.767	0.00	0.46	0.503	0.01
	sleep*practice	0.22	0.638	0.01	0.00	0.975	0.00

This table presents uncorrected p-values for all the results of mixed-model ANOVAs performed on the

behavioral parameters. Between-subject factors were sleep (Wake, Sleep) and practice (Random, Blocked). The

within-subject factor was time changes (Adaptation: First Training Trials, Last Training Trials; Consolidation:

Last Training Trials, Posttest; Generalization: Last Training Trials, Transfer). For adaptation of the adaptation

coefficient, the factor time contained error clamp trials at Baseline and the end of Training. Effect size is the partial

eta squared (pη²); p-values < 0.05 are in bold.

The Random and Blocked groups started with similar Posttest performance (Fig. 2a,b), showing that consolidation from the Last Training Trials (11 hours earlier) to the Posttest was worse for the Blocked groups than the Random groups (Fig. 2b), but independent of sleep in any of the groups. An uncorrected supplementary per-trial analysis suggested the benefit of Random over Blocked training appeared quickly during the Posttest (Supplementary Fig. S1). This benefit was confirmed by uncorrected Pearson correlations showing that training success (i.e., lower motor error at the end of Training) was inversely related to consolidation success (percentage of Posttest error related to last Training error (*r* = −0.78, *p* < 0.001, *n* = 48), with a weaker effect in the Random groups (*r* = −0.51, *p* = 0.01, *n* = 24) than the Blocked groups (*r* = −0.86, *p* < 0.001, *n* = 24; *z* = 2.36, *p* = 0.018, for the difference using Fisher’s r-to-z transformation). However, as expected, individual levels seemed stable as training success, in general, was still moderately predicted and positively correlated with overall Posttest performance of all the groups (*r* = 0.29, *p* = 0.044, *n* = 48; *r* was within 0.17–0.35 for all the groups). This suggests that motor-memory retention benefits from a randomized training schedule even though initial Training performance might benefit from a blocked training schedule. These processes were not affected by sleep.

We further investigated whether memory consolidation enhanced generalization from the dominant hand (during Training) to the non-dominant hand (Transfer). All groups performed more poorly during Transfer compared to the Last Training Trials. In addition, the initial Transfer performance of all the groups was worse compared to their initial Training performance (First Training Trials, Fig. 2). This lower initial performance during contralateral transfer learning indicates that participants expected the force field would be in the opposite direction, as it was during Training (relying on an internal rather than an external representation). This is supported by the adaptation coefficient (the force-field compensation factor), which showed similar force-field predictions during the initial Transfer trials of the Blocked and Random groups (Blocked: −15.64%, *SD* = 16.61%; Random: −15.62%, *SD* = 17.85%; the negative sign indicates expectation of an opposite force-field direction). Motor error in the Transfer test was lower in the Random groups than the Blocked groups and this effect is indicated to appear immediately after the first Transfer trial (Fig. 2a, Supplementary Fig. S1). Transfer learning effects were independent of sleep and did not strongly predict training success (all groups: *r* = 0.12, *p* = 0.42, *n* = 48; Random groups: *r* = 0.14, *p* = 0.54, *n* = 24; Blocked groups: *r* = 0.40, *p* = 0.056, *n* = 24). However, transfer learning was strongly influenced by motor-memory consolidation; that is, improvements over the consolidation period from the Last Training Trials to the Posttest were associated with improvements from the Last Training Trials to Transfer (*r* = 0.84, *p* < 0.001, *n* = 48, for uncorrected Pearson correlations); the association was weaker for the Random groups (*r* = 0.48, *p* = 0.024, *n* = 24, for uncorrected Pearson correlations) than the Blocked groups (*r* = 0.88, *p* < 0.001, *n* = 24; *z* = 2.72, *p* = 0.007, for the difference using Fisher’s r-to-z transformation). This suggests, in general, that enhanced consolidation from Training to Posttest is strongly related to enhanced Training to Transfer consolidation, but transfer learning was impeded less by motor-memory consolidation after random training than after blocked training.

**Figure 3:**
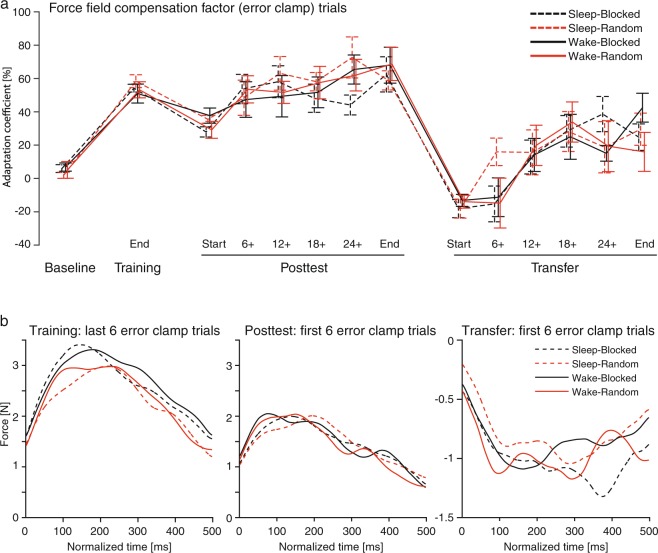
(**a**) Progress of the force-field compensation factor measured by the mean (±S.E.M) adaptation coefficient in error clamp trials for the Blocked (black) and Random (red) groups during all Baseline trials, the last 6 Training trials (End), Posttest trials (averaged across blocks of 6 trials at Start/End and 4 interspersed trials (6+, 12+, 18+, 24+), and Transfer trials (averaged across blocks of 6 trials at Start/End and 4 interspersed trials). (**b**) Corresponding mean force over the trial duration (normalized to 500 ms) for the last 6 Training trials, first 6 Posttest and Transfer trials over all four groups.

Motor error, quantified by EA, was affected by both predictive feedforward and responsive motor feedback. As feedback responses typically start to compensate for feedforward errors at 100 ms^30^, and the average trial duration across groups was about 550 ms in the current study, EA should mostly reflect the feedback responses. Thus, we tested if the observed effects of training conditions also underlied feedforward motor predictions measured by the adaptation coefficient (Fig. 3a,b). Neither the training nor the sleep conditions influenced adaptation coefficient changes from Training to Posttest or from Training to Transfer (Table 1). This indicates that the effect observed here is influenced more by late feedback than early feedforward responses which is also indicated by other behavioral parameters (see Supplementary Table S2).

### Task-EEG

Exploratory analyses, using cluster-based statistics, were conducted to test possible retention*sleep effects (with retention: Last Training Trials, Posttest; Last Training Trials, and Transfer). The analyses revealed that cortical activity in all the frequency bands were unaffected by Sleep vs. Wake. Thus, we focused on further task-EEG analyses regarding the training conditions (Blocked vs Random).

**Figure 4:**
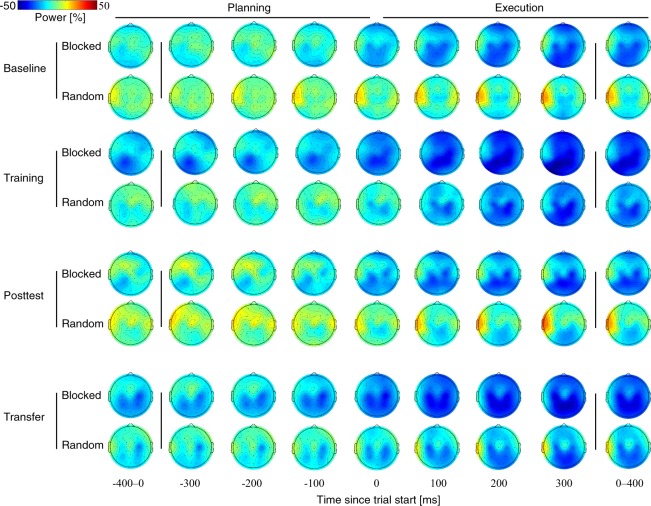
Progress of mean alpha-band power for Blocked and Random groups during the Training session and the Posttest and Transfer tests of the Retest session. The leftmost and rightmost topographies represent mean power across motor planning (−400–0 ms) and execution (0–400 ms) with respect to the start of the trial (0 ms). Other plots represent topographical power at specific points in time (from −300 ms to 300 ms). Power values are percentages of the average reference period 250 ms before highlighting of the fixation cross.

**Figure 5:**
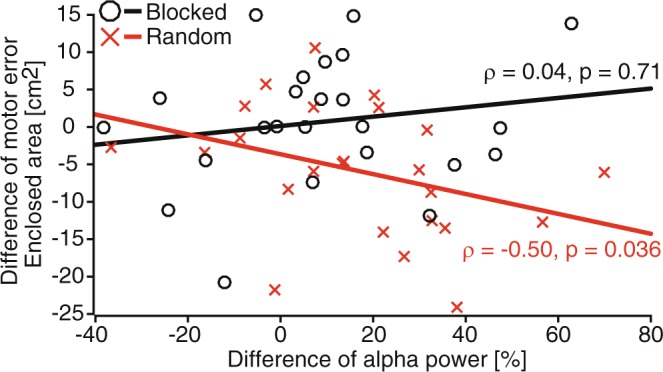
Associations between Training-to-Posttest difference in motor error (quantified by the enclosed area) and alpha power (8–13 Hz) for ROIl (CP5, CP1, Pz, and P3) during motor execution were tested using Spearman’s correlation. Each red cross represents the data of a single participant from the Random group and each black circle represents a participant from the Blocked group. Lines represent a basic linear fit (red: Random; black: Blocked) and *ρ* represents Spearman’s rho with associated *p*-values.

Analysis of a possible training condition effect was restricted to the alpha-band power (Fig. 4) over parietal areas, based on previous research that showed a link between training effects and force-field adaptation only in the alpha frequencies^27^. Given these previous findings, we defined a left- and right-hemispheric region of interest (ROIl: CP5, CP1, Pz, and P3; ROIr: CP6, CP2, Pz, and P4). The analyses found significantly higher alpha-band power for the Random groups compared to the Blocked groups on the Posttest during movement execution, and a similar effect, which did not reach significance, on the Transfer test [Posttest, *t*(46) = −2.22, *p* = 0.031, *d* = 0.642, for ROIl; Transfer, *t*(44) = −1.85, *p* = 0.072, *d* = 0.543, for ROIr). Increased alpha-band values over ROIl from Training to the Posttest, during movement execution, were associated with better task-consolidation success (quantified by a small Training-to-Posttest difference in motor error; Fig. 5) in the Random groups but not the Blocked groups (Random, *ρ* = −0.50, *p* = 0.036; Blocked, *ρ* = 0.04, *p* = 0.710, for FDR corrected Spearman correlations, with *ρ* representing Spearman’s rho). This suggests that random training of force fields affected parietal alpha-band activity on the Posttest.

**Figure 6:**
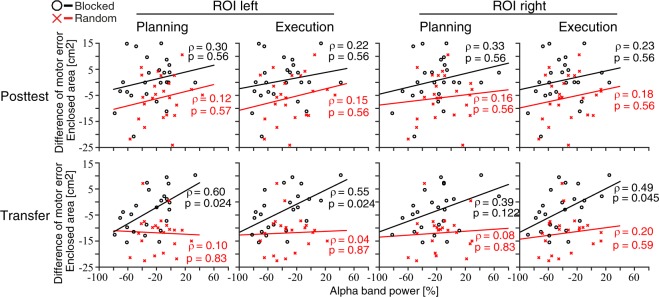
Prediction of motor-memory consolidation (Training-to-Posttest or Training-to-Transfer difference in motor error) by alpha power (8–13 Hz) during Training. Each plot represents the data points for each participant of the Blocked (black circle) and Random (red cross) groups together with their groups’ linear fit. Lines represent basic linear fit, and *ρ* represents Spearman’s rho with associated FDR-corrected *p*-values (across 8 values of groups, phases, and ROIs).

Furthermore, we explored whether the behavioral retention effects (Training-to-Posttest, Training-to-Transfer) were predicted by EEG’s alpha-band power during Training. Spearman correlations revealed positive but weak associations from Training-to-Posttest for both groups (Random and Blocked) phases (planning, and execution) and ROIs (ROIl and ROIr), which did not reach statistical significance (Fig. 6). On the other hand, the associations from Training-to-Transfer consolidation were strong for the Blocked groups (all *ρ* between 0.39 and 0.60), but still weak for the Random groups (*ρ* between 0.04 and 0.20). These positive correlations for the Blocked groups were still statistically significant after FDR correction (across 8 values of groups, phases, and ROIs) for ROIl (Fig. 6: planning, *ρ* = 0.60, *p* = 0.024; execution, *ρ* = 0.55, *p* = 0.024), but only during trial execution for ROIr (planning: *ρ* = 0.39, *p* = 0.122; execution, *ρ* = 0.49, *p* = 0.045; the uncorrected *p*-values are presented in Supplementary Table S3).

### Sleep-EEG

Though overnight sleep during consolidation did not improve motor performance more than daytime wakefulness, we examined which activity during sleep might indicate the consolidation processes that occur during sleep. None of the basic sleep-stage parameters was correlated with consolidation performance (Supplementary Table S4).

Consolidation success was predicted by sleep spindles and their occurrence during upstates of slow waves (Supplementary Table S5). Longer sleep spindles and, especially, their occurrence (their count and density) during upstates of slow waves, were associated with improvements from Training to Posttest and Training to Transfer. This effect was most pronounced in the left parietal area (i.e., P3, corresponding to ROIl) and indicated by more slow-wave activity (power density) during sleep-spindle enriched Stage 2 sleep. A moderate decline in the oscillation frequency (i.e., less “chirp” toward lower oscillation frequencies) during such spindles predicted a deterioration from Training to Transfer, whereas a higher sleep-spindle oscillation frequency in fronto-central areas predicted a deterioration of motor performance from Training to Posttest and Training to Transfer. Higher dispersion of sleep spindles locked to the slow-wave down state (in Cz), which generally suggests impaired mechanisms involved in the timing of sleep spindles and slow waves, predicted less improvement during Transfer compared to Training. No other sleep measures were significantly correlated with consolidation measures.

## Discussion

Our results showed that the Random and Blocked groups adapted to the force-field conditions successfully. Subsequent consolidation was influenced by training conditions but not intervening sleep. Although Training outcomes were worse in the Random than the Blocked groups, all the groups showed similar motor performance on the Posttest, and the Random groups had more pronounced motor performance compared to the Blocked groups, when tested for Transfer on the untrained, non-dominant hand. This improvement was expressed by reductions in motor error (enclosed area), which is mostly affected by response feedback corrections, but not by the adaptation coefficient (force-field compensation).

Our behavioral results found no significant effect of sleep on the motor adaptation task. Task performance and measures of consolidation were independent of whether participants were awake or asleep during the intervening time between Training and Retest. Thus, our study confirms earlier findings^5,16^ and are consistent with the findings of some force-field studies^17,18^ demonstrating that the consolidation of motor adaptation to dynamic perturbations depends on time, but not sleep. Previous research suggested that force-field adaptation represents implicit memory that does not depend on the hippocampus^17,31^. Thus, our negative findings agree with the assumption that only hippocampal-dependent motor processes benefit from sleep^1^. However, research has shown that hippocampal damage decreases the benefits of variable training conditions^25^, indicating that a motor task might become hippocampal-dependent, and thus, would be expected to become sleep-dependent when trained under highly variable conditions^1,22^. However, this view is not supported by our behavioral data, which also failed to show any sleep effects for the Random groups. The exact extent to which motor adaptation after variable training becomes hippocampal-dependent is unclear and needs to be investigated in future studies.

Despite the lack of evidence of a consolidation benefit of sleep, compared to wakefulness, on retention, consolidation success correlated positively with sleep spindle activity during slow-wave upstates. Though we cannot exclude the possibility this reflects spurious correlations, this surprising finding is at odds with the view that consolidation of motor adaptation is completely independent of hippocampal processes, because the coalescence of spindle and slow-wave activity during sleep is thought to enhance consolidation, particularly in hippocampal-dependent tasks^31–33^. Intriguingly, we found alpha activity to be associated with task-consolidation across parietal brain regions, which coincides with the strong association of sleep-mediated consolidation in the same regions. This concurs with the view that cortical regions that are engaged in learning have a strong local association with spindles and slow waves in subsequent sleep^34^ and predict the extent of consolidation^14^. Whether such associations were functionally involved in the consolidation process in our study, or merely reflect the consolidation success of other memories that were not tested by our task, remains unclear.

We found variable training in a motor adaptation task predicted consolidation benefits. This study, therefore, reproduced earlier findings of the contextual interference effect^21,35^, in that higher training variability led to decreased motor performance at the end of Training that was similar to that of blocked-training on the Posttest, and comparable to performance benefits on the Transfer test. As previously reported^27^, this induced effect of variable training is only seen in motor error, which is mainly influenced by feedback responses. In contrast, the adaptation coefficient (and the kinematic prediction angle; see Supplementary Table S2) did not exhibit this effect. In fact, the adaptation coefficient and the kinematic prediction angle showed similar results for all main effects and interactions. Because the participants did not receive task-specific feedback during error clamp trials, the adaptation coefficient was solely feedforward-dependent^28,29^. Therefore, it is likely that the motor benefits in the Random groups were facilitated by feedback corrections during movement execution that were evoked by the permanent regulation of random, unexpected forces during Training.

Although the performance of the Random groups at the end of training was worse than that of the Blocked groups, their Posttest performance was comparable. This suggests that variable training either primes better memory consolidation or forms memories that are more stable. In addition, generalization to the Transfer test on the left hand was more pronounced in the Random groups. The consistency of this benefit on generalization over 30 trials speaks to a stable long-term memory effect. This was confirmed by the finding of a significant association of Training performance and benefits on memory consolidation, which shows that a lower Training performance (in terms of higher motor error), resulting from variable training, also led to better retention performance.

During Transfer testing, the participants expected a force field on their left hand that was in the opposite direction of the force field they experienced while Training the right hand. This explains the initially poorer Transfer performance of all the groups compared to their initial Training and Posttest performance. This suggests the generalization did not take place in an extrinsic force-field transformation, but through intrinsic, mirror symmetric coordinates, which contradicts previous findings^36^. Paradoxically, we did not find an even greater decrease in initial Transfer performance in the Random groups, as would be expected for a more consolidated intrinsic representation in this group, which gives rise to predicting a force field in the opposite (wrong) direction during Transfer. However, the opposite was the case, i.e., the Random groups showed enhanced Transfer performance compared to the Blocked groups. There are three possible explanations for this outcome.

First, generalization was worse for the Random groups compared to the Blocked groups. The increased motor performance of the Random groups might be facilitated by a weaker generalization or consolidation of the generalized memory. However, motor performance, quantified by the adaptation coefficient, showed similar Transfer performance in all the groups, indicating they all had similar generalization.

Second, random training favored the formation of memory in a different coordinate system (extrinsic, intrinsic, or a mixture of systems^37,38^). The results, however, do not support such an explanation, as the adaptation coefficient was similar among the groups. In addition, inspection of individual data revealed cues for an extrinsic force-field representation in only 4 of the 24 participants in the Random groups. This was also the case for 2 of the 24 participants in the Blocked groups.

Third, random training led to a generally increased ability to use feedback responses. This explanation is supported by the finding that only motor error, which is sensitive to feedback corrections, but not the adaptation coefficient, showed increased memory consolidation in the Random groups. In addition, the EEG data showed that parietal, but not frontal, areas of the brain were involved in the contextual interference effect, with the former known to be specifically implicated in sensory integration^39^. However, future research should further investigate the influence of variable training on online feedback corrections in motor behavior.

Summarizing the behavioral findings, variable training led to benefits in consolidation of a force-field adaptation task. This effect was more prominent when retention was tested on the contralateral hand. We assume that the increased consolidation after highly variable training was facilitated by an increased ability to use online feedback corrections.

The task-EEG during task performance showed that behavioral changes across the consolidation period after Random training was accompanied by a parallel increase (from Training to Posttest) in alpha-band power over parietal areas, which agrees with previous findings from our laboratory^27^. Specifically, we were able to reproduce a negative correlation between changes in alpha power over contralateral parietal areas (ROIl) and motor error during movement execution. An increase in alpha-band power has frequently been mentioned as a sign of active inhibition of underlying cortical regions^40^. Therefore, a negative correlation might indicate that more accurate, and thus, better consolidated motor performance occurred in parallel with increased inhibition of parietal areas.

The results also showed that consolidation in this force-field adaptation task was predicted by alpha power over parietal areas during Training. Blocked groups, but not Random groups, showed significant associations between Training-to-Transfer consolidation and alpha-band power. Therefore, high parietal alpha-power and inhibition of parietal cortical areas during Training might have favored weaker consolidation for the Blocked groups but not the Random groups. The questions arising here are whether the greater efficacy of random training specifically results from its ability to counter the disadvantage of increased parietal alpha power during training or whether parietal alpha power is connected to online feedback corrections of the motor system.

The behavioral parameter used to measure motor error (EA) does not accurately detect motor adaptation. Over the years, several other parameters have been developed that are used instead of EA. Though we are aware of this limitation, the EA parameter has some advantages that are important for this study. EA equates more closely with feedforward mechanisms and motor performance because it accounts for the whole trajectory. Other parameters, such as the maximum perpendicular displacement or the perpendicular displacement at peak velocity, compute the perpendicular displacement only at a certain point in time. Moreover, this point in time shifts due to adaptation, consolidation, and generalization. This time shift leads to different percentage time amounts of feedforward contributing to the parameter in each single trial. To ensure our findings were not affected by choosing the EA parameter, we demonstrated that our results can be reproduced with other parameters (Supplementary Table S2). Another reason that EA is a better choice for computing associations with EEG signals is that those signals are averaged over almost the entire trial.

Another limitation of our behavioral findings is that two out of 6 parameters, which were used in the study (Table 1 and Supplementary Table S2), show a disadvantage of having sleep on the generalization (although the effect is weak in both cases). Therefore, we cannot rule out possible confounding effects of sleep on motor memory generalization. In addition, this study lacks testing of the transfer performance on the non-dominant hand against a baseline performance of the left hand. However, we expect this has not affected our results given that a previous study^41^ showed the baseline performances between hands do not differ much.

## Conclusion

Sleep does not contribute more to the consolidation than being awake does in motor-memory force-field adaptation tasks. However, training variability affects motor-memory consolidation and generalization to the contralateral hand. This increase in retention produced by random training is associated with parietal alpha frequencies in the left hemisphere and might be influenced by individual differences in feedback mechanisms. A remaining question is whether this activity over parietal regions is connected to online feedback corrections of the motor system. Furthermore, it remains an open question why sleep did not aid consolidation behaviorally, although explorative analyses suggested a correlation of consolidation with sleep spindles that were nested in slow-wave upstates over parietal sites, i.e., indicators of hippocampal-dependent memory processing during sleep at learning-specific cortical sites.

## Methods

### Participants

Forty-eight healthy, male participants, recruited from a local university campus, were included in the study (age 24.27 ± 0.45 years). All the participants were native German-speakers with normal or corrected to normal vision and were tested for right-handedness using the Edinburgh handedness inventory^42^. They reported no sleep disorders or habitual napping. Participants did not take any medication at the time of the experiment and did not drink coffee habitually. Participants followed a normal sleep–wake rhythm and reported no night shifts during the 6 weeks before the experiment. Participants were instructed to keep a regular sleep schedule, abstain from caffeine- and alcohol-containing drinks for at least 2 days before and on the days of the experiment. The experimental task and task-protocol were new to the participants. All participants provided written informed consent to be in the study, and in relevant cases, for publication of identifying images in an online open-access publication. The study was approved by the ethics committees of the Karlsruhe Institute of Technology and the University of Tübingen. All the methods were conducted in accordance with the relevant guidelines and regulations.

### Apparatus and motor adaptation task

The apparatus and task were adapted from a previous study (see^27^, for a detailed description). Participants performed point-to-point reaching movements with a robotic manipulandum (Kinarm End-Point Lab, BKIN Technologies, Kingston, Canada; Fig. 1a). The manipulandum measured the position of the handle and forces exerted on the handle at 1000 Hz. Participants grasped the handle with their forearm supported by an air-sled system that enabled low-friction movements. The goal of the task was to move a cursor on a screen – controlled via the robot handle – into a target circle (Fig. 1b). To prevent movement anticipation, each trial started with a fixation cross that randomly varied in duration between 0.8 and 1.5 s. When the fixation cross changed its shape to a target circle, participants were allowed to initiate movement (no fast reaction times were required). After reaching the target, the manipulandum actively guided the subjects’ hands back to the center point, at which time the next trial was begun. In total, six targets were arranged in a circle surrounding the center target. The distance between each of the six targets and the center target was 10 cm and the diameter of each target was 0.45 cm. The target order was pseudo-randomized so that every target was highlighted just once in every block (containing 6 movements). Within each group, the target order was different for every subject, so that the mean target direction and the mean force-field magnitude across all subjects were identical for each trial.

The manipulandum produced forces that were applied to each subject’s hand via the handle. This study used three types of trials: null-field trials, force-field trials, and error clamp trials. In null-field trials, no forces were produced and participants performed movements under undisturbed conditions. In force-field trials, the motors of the manipulandum were turned on to produce a velocity-dependent curl force-field in a clockwise direction with three magnitudes: 10, 15, and 20 Ns/m. In error clamp trials, the manipulandum produced a virtual force-channel from the start point to the target so that the subjects were only able to move along this path directly into the target (Fig. 1c). On every trial, visual feedback about the movement time was given to ensure similar movement times across trials and subjects (<450 ms: too slow; >550 ms: too fast).

Offline calculations of the behavioral dependent variables were performed using MATLAB R2015b (MathWorks Inc., Natick, MA, United States). For null-field and force-field trials, we computed motor error using the enclosed area (EA) between subjects’ hand path and the vector joining the start point and the target (Fig. 1c, left). This parameter was averaged over 30 trials for Baseline, First Training Trials, Last Training Trials, Posttest, and Transfer. To quantify motor performance on error clamp trials, we calculated a force-field compensation factor (Fig. 1c, right) using linear regression on the measured and the ideal perpendicular force profiles^28^ and averaged this across each of the 6 error clamp trials. Regression analysis was done by using the polyfit function in MATLAB with a degree of 1 (Supplementary Methods). The first coefficient of the linear component was used for computing the compensation factor with the constant term not forced to pass through zero. As participants did not receive error-feedback during these trials, this parameter mainly reflected movement predictions, and thus, feedforward mechanisms^29^. In this study, the term motor error refers to the enclosed area and the term adaptation coefficient refers to the force-field compensation.

### Design and Procedures

This study compared the effects of Random (high variability) vs. Blocked (low variability) training on motor adaptation and consolidation processes during overnight sleep (Sleep) versus daytime wakefulness (Wake). Participants were randomly assigned to four equal-size groups (*n* = 12) of comparable age (range = 18–30 yrs; *p* > 0.45), in a one-way, between-group ANOVA design, with altered training conditions and retention periods occurring either during the night or during the day. All participants were trained with their dominant right hand on a motor adaptation task; the groups differed only in the schedule of force-field magnitudes. The task was either trained in a random sequence of force-field magnitudes (Random group) or in three randomized blocks, each containing a consistent field magnitude (Blocked group). Participants either trained in the morning (9 am; Fig. 1d) and retested in the evening (8 pm; Wake-Random, WR; Wake-Blocked, WB), or trained in the evening and retested the following morning after a night of sleep (Sleep-Random, SR; Sleep-Blocked, SB). The retention period between the Training and the Retest sessions was about 11 hours for all the groups. The Wake participants spent their waking time engaged in their usual daily activities and the Sleep participants went home after Training to sleep, while polysomnographic recordings were made. The retest session contained a Posttest and Transfer test, which quantified the motor performance of participants using their right hand (Posttest) and left hand (Transfer) (Fig. 1d).

Before Training, participants familiarized themselves with the motor adaptation task and EEG recordings. During Familiarization, participants performed 144 null-field trials with their right hand. Before Training and the Posttest, participants were tested for the possible confounding effects of subjective sleepiness (the Stanford Sleepiness Scale, SSS^43^), mood (the Positive Affect Negative Affect Scale, PANAS^44,45^), and objective vigilance (a 5-min Psychomotor Vigilance Task, PVT^46^).

Then, participants provided Baseline data by performing 30 null-field trials and 6 error clamp trials. Training consisted of 144 force-field trials followed by 6 consecutive error clamp trials. All the participants were trained on force-field trials that were divided into three force-field magnitudes (10, 15, and 20 Ns/m), with a mean force-field magnitude of 15 Ns/m over all the trials. The Random and Blocked groups trained with the magnitudes under different training schedules that manipulated the training variability of those groups: the Random group was trained on all the trials with the force-field magnitude switching from trial to trial in a pseudo-random order (high variability); the Blocked groups were trained in three blocks of trials, each of which contained 48 trials with consistent force-field magnitudes; the force-field magnitudes were only switched between the blocks. The block order was counterbalanced across the participants in each group. When the Training session for the Wake group participants ended, they were given instructions for their arrival for the Retest session in the evening. The Sleep group participants received additional instructions for the overnight home-polysomnography recording. The Sleep group started the Retest session with the removal of the sleep-EEG equipment.

The Retest session was the same for all the participants: they performed a Posttest of the task with 6 error clamp trials, 30 force-field trials, and 6 error clamp trials. In addition, the 30 force-field trials were interspersed with an error clamp trial after every 6 force-field trials. All the force-field trials were fixed at the mean force-field magnitude of the Training (15 Ns/m). The Posttest was followed by the Transfer test. The Transfer test used the same protocol as the Posttest and participants performed the behavioral task with the non-dominant left hand. Note that the force-field direction in the Transfer test was still clockwise.

### Task-EEG

To record EEG during task performance, we used the actiCHamp system with 32 active-electrodes, and we used the BrainVision PyCorder (V1.0.6) for data recordings (Hardware and software from Brain Products, Gilching, Germany). The task-EEG was synchronized with the manipulandum via a direct link and the data were sampled at 1000 Hz. A cap was placed on the subject’s head containing 29 electrodes that recorded cortical activity using the international 10–10 system (Fp1, Fp2, F7, F3, Fz, F4, F8, FC5, FC1, FC2, FC6, T7, C3, Cz, C4, T8, CP5, CP1, CP2, CP6, P7, P3, Pz, P4, P8, TP10, O1, Oz, and O2). The remaining three electrodes were used to record horizontal and vertical eye movements. Electrode Cz was used as the reference and Fpz was used as the ground electrode. The impedances of the electrodes were kept below 10 kΩ.

Offline EEG analyses were performed using MATLAB R2015b (MathWorks Inc., Natick, MA, United States) and EEGLAB 13.5.4b^47^. The raw data of the task-EEG were filtered first by a FIR high-pass filter with a cut-off frequency of 0.5 Hz and then by a FIR low-pass filter with a cut-off frequency of 281.25 Hz. Line noise was removed using the cleanline plugin for EEGLAB. Channels strongly affected by artifacts were removed by visual inspection and the missing channels restored using spherical interpolation. Electrodes were re-referenced to the average reference and channel location Cz was reconstructed and appended to the data. Then, the EEG data were divided into epoch segments of 8.5 s, ranging from 6 s before to 2.5 s after the start of each trial. Principle component analysis (PCA) was performed to compress the data to 99.9% of the variance, and thus, deal with the reduced rank due to interpolation. Then, infomax independent component analysis (ICA^48^) was performed on the principle components. To detect bad ICA components, the components were evaluated in the spectral, spatial, and temporal domains. Components showing distinct artifacts were rejected and the data were re-transformed into the channel domain.

We calculated the percentage power in the frequency domain for subsequent statistical comparisons, using complex Morlet wavelets for the frequency decomposition. We decomposed the data into 40 frequency bins, ranging from 2 to 100 Hz, in a logarithmic space of 5 to 19 wavelet cycles changing as a function of frequency. The decomposed data were averaged over 30 trials and squared, resulting in the average power for Baseline, First Training Trials, Last Training Trials, Posttest, and Transfer. Then, power was normalized according to the average reference period of 250 ms before the highlighting of the fixation cross, and the event-related desynchronization/synchronization (ERD/ERS) was calculated^49^.

Data were averaged in the frequency domain into specific frequency bands: theta (4–7 Hz), alpha (8–13 Hz), beta (14–30 Hz), and gamma (30–45 Hz). The data were also compressed in the time domain by averaging two time windows: movement planning (−400 ms to 0 ms) and movement execution (0 s to 400 ms), where 0 indicates the start of the trial.

### Polysomnography and sleep-EEG analyses

Standard polysomnography was measured using a home-recording system (Somnoscreen Plus, Somnomedics, Randersacker, Germany), with EEG at locations F3, F4, Fz, C3, C4, Cz, P3, P4, and Pz (International 10–20 system). The electrooculography (EOG) sites were around the eyes, and electromyography (EMG) was measured with electrodes placed at each musculus mentalis and two electrodes placed at each mastoid behind the ear. Fpz served as the ground electrode and Cz served as the original reference. The data were digitized at 256 Hz and down-sampled to 128 Hz to facilitate computation. Offline manual sleep scoring and automatic basic sleep-EEG analysis were performed using the open-source toolbox SpiSOP^50^. Data from two participants (one from the Blocked group and one from the Random group) were excluded from the analyses due to technical failures (n = 22). Scoring was performed by an experienced rater, blind to the participants’ conditions, according to standard criteria^51^. Sleep-EEG analyses, apart from sleep scoring, were performed on EEG channels re-referenced to the average signals from the mastoids. Sleep-EEG parameters were detected using standard settings of SpiSOP^50^, based on the analyses described in Wang *et al*.^52^ and briefly described below.

#### Power spectral analyses of sleep EEG

Power spectra were calculated separately for Stage 2, SWS, non-REM, and REM sleep on consecutive artifact-free 10-s intervals of non-REM sleep, which overlapped in time by 9 s. Each interval was tapered by a single Hanning-adapted window (1 s tails following the Hanning window, the other 8 s were 1) before applying a Fast Fourier Transformation that resulted in interval power spectra with a frequency resolution of 0.1 Hz. Power spectra were, then, averaged across all the blocks (Welch’s method) and normalized by the effective noise bandwidth to obtain power-spectral density estimates for the entire data. The mean power density in the following frequency bands was determined: slow-wave activity (0.5–4 Hz), theta (4–8 Hz), spindles (9–15 Hz), alpha (8–12 Hz), slow spindles (9–12 Hz), fast spindles (12–15 Hz), and beta (15–30 Hz). Frequency bands were log transformed (decibels) prior to statistical testing.

#### Slow waves

For the identification of slow waves, the signal in each channel during non-REM sleep epochs was filtered between 0.5 and 3.5 Hz. Next, all the time intervals with consecutive positive-to-negative zero crossings were marked as putative slow waves if their durations corresponded to frequencies between 0.5 and 1.11 Hz (zero crossings marked the beginning and end of slow oscillations), but these were excluded if their amplitudes were >1000 mV (as these were considered artifacts) or when both negative and positive half-wave amplitudes were between −15 and +10 mV. A slow wave was identified (a) if its negative half-wave peak potential was lower than the mean negative half-wave peak of all putatively detected slow oscillations in the respective EEG channel, and (b) if the amplitude of the positive half-wave peak was larger than the mean positive half-wave amplitude of all other putative slow waves within the channel. For each participant and channel, the number of slow oscillations, their density (per min non-REM sleep), mean amplitude, and slopes (down slope = the ratio between the value of the negative half-wave peak and the time to the initial zero crossing; up slope = the ratio between the absolute value of the negative half-wave peak and the time to the next zero crossing) were calculated.

#### Sleep spindles

For each EEG channel, the signal during non-REM epochs was filtered in a 2-Hz frequency band centered to the visually determined corresponding power peak (12 to 15 Hz range, 13.32 ± 0.11) in the non-REM power spectrum of each participant. Then, the root mean square was computed using a sliding window with a size of 0.2 s, and the resulting signal was smoothed in the same window with a moving average. A sleep spindle was identified when the smoothed RMS signal exceeded an individual amplitude threshold by 1.5 standard deviations of the filtered signal in the channel at least once for 0.5 to 3 s. The threshold crossings marked the beginning and end of each spindle and quantified their duration. Sleep-spindle amplitude was defined by the voltage difference between the largest trough and the largest peak. Spindles were excluded with amplitudes >200 µV. We focused the analysis only on fast spindles, as power peaks of slow spindles could not be clearly identified in too many participants. Absolute spindle counts, spindle density (per min non-REM sleep), mean amplitude, average oscillatory frequency, and duration were calculated for each participant and channel.

#### Sleep spindles co-occurring with slow-wave upstates

To explore if spindles co-occurring with slow waves possessed properties that were associated with behavior, we identified slow waves that had at least one detected sleep spindle from the lowest trough (down state) to +0.5 seconds after the next positive-to-negative zero crossing (i.e., slow-wave upstate). To avoid duplicate matches, sleep spindles were counted only once for the first slow wave in which they occurred within the same channel. Then, the properties of these co-occurring sleep spindles and slow waves were determined as mentioned above. In addition, the mean delay of sleep spindles to the slow-wave down state and the standard deviation of this delay were calculated to estimate the temporal dispersion of their co-occurrence.

To conduct an exploratory analysis of standard and fine-tuned sleep EEG parameters and their associations with memory consolidation, power density, slow waves, and sleep spindle parameters (e.g., density) were averaged per electrode. Pz, F3, and F4 were excluded from the analysis of two sleep subjects and C4 was excluded in one sleep subject, as these electrodes fell off during sleep EEG recordings with otherwise good sleep EEG.

### Statistical analysis

We used independent, two-tailed *t*-tests and mixed-model ANOVAs with the within factors time (First Training Trials, Last Training Trials) and retention (Last Training Trials, Posttest, Last Training Trials, Transfer), and the between factors sleep (Sleep, Wake) and training (Random, Blocked) to test differences in motor error (EA) and the adaptation coefficient (force-field compensation). Data normality was tested using the Shapiro-Wilk *W*-test, and parametric or nonparametric statistical tests were used accordingly. The assumption of equal group variances was tested using Levene’s test to choose the appropriate *t*-test.

Statistical analyses of task-EEG possibly related to sleep effects were performed using cluster-based statistics corrected for the maximum permuted cluster values^53^. Therefore, mixed model ANOVAs, with the factors retention (Last Training Trials, Posttest; Last Training Trials, Transfer) and sleep (Sleep, Wake), were performed for every frequency band during movement planning and execution. Clusters were computed at the channel level according to the *p*-values of the ANOVAs and the summed *F*-value for each cluster was stored as the observed statistic. Then, permutation testing was done using 10,000 iterations. For each iteration, the data were mixed across both dimensions (retention, sleep), the ANOVA was computed, and the maximum cluster value was stored. Each *p*-value was defined as the sum of maximum permutation clusters exceeding the observed statistic divided by the number of iterations.

Furthermore, we tried to reproduce previous findings from our laboratory^27^ targeting the neural basis for the benefits of variable training. Accordingly, independent *t*-tests between training groups (Random, Blocked) were performed to test alpha-band power differences during Posttest and Transfer. In addition, motor error differences between Posttest and Last Training Trials (Training-to-Posttest) and Transfer, and Last Training Trials (Training-to-Transfer) were computed for each participant at the behavioral level and correlated with the EEG data during Training, using Spearman’s correlations.

Likewise, to find potential correlates of consolidation success with sleep parameters, we performed an explorative analysis across both Sleep groups using Spearman’s correlations between behavioral changes over the retention period and all the sleep parameters [i.e., total sleep time (TST); sleep onset delay; duration and percentage of TST sleep during stages, such as being awake after sleep onset, Stage 1, Stage 2, SWS, non-REM (i.e., SWS + Stage 2); the power density of each sleep stage in the prominent frequency bands; the parameters of slow waves, sleep spindles, and their co-occurrence during the slow-wave upstates]. Due to the explorative nature of the analyses in the absence of a behavioral sleep effect, we did not correct these correlations for multiple comparisons.

Statistical analyses were performed using MATLAB R2015b (Mathworks, Natick, USA) for Windows, JASP 0.8.6^54^, and R [Windows 64 bit version, 3.3.1, R Development Core Team, The R Foundation for Statistical Computing. 2007 (www.r-project.org/foundation)]. The threshold for statistical significance was set at *p* = 0.05. Multiple comparisons were either corrected by the maximum statistic (permutation test) or by the False Discovery Rate (FDR^55^). In the case of FDR, multiple comparisons were done at the post-hoc test level. Please note that adaptation, consolidation, and generalization target different research questions which is why we did not correct *p*-values across these three computations. The *p*-values testing the main hypotheses of this study are presented by FDR-corrected *p*-values explicitly mentioned as such^56^. For simplicity, confirmatory, explanatory, and exploratory analyses report uncorrected *p*-values. Effect sizes were determined using partial eta squared (*pη²*) and Cohen’s *d*.

## Electronic supplementary material

Supplementary Information

Supplementary Dataset

## Data Availability

All data needed to evaluate the conclusions in this paper are presented in the paper and/or the Supplementary Materials, including a Supplemental Data file (csv format) containing all the individual data points. Additional data related to this paper may be requested from the authors. The computer codes used to generate the results will be provided for free upon request. The codes for the sleep analyses and the standard parameters are publicly available at www.spisop.org.
