# Supplementary material for: Variable training but not sleep improves consolidation of motor adaptation
Source: Sci Rep. 2018 Oct 29;8:15977. doi: 10.1038/s41598-018-34225-w (PMC6206141; doi:10.1038/s41598-018-34225-w)
Supplement: Supplementary file 1 — Supplementary Information [file 41598_2018_34225_MOESM1_ESM.pdf]

Supplementary Information

Variable training but not sleep improves consolidation of motor adaptation

Benjamin Thürer[1*†], Frederik D. Weber[2,3*†], Jan Born[2 ‡], Thorsten Stein[1 ‡]

[1]BioMotion Center, Institute of Sports and Sports Science, Karlsruhe Institute of Technology, 76131 Karlsruhe, Germany

[2]Institute for Medical Psychology and Behavioral Neurobiology, University of Tübingen, 72074 Tübingen, Germany

[3]Donders Institute for Brain, Cognition, and Behaviour, Radboud University Medical Centre, 6525 EN Nijmegen, the Netherlands

*Corresponding authors: benjamin.thuerer@gmail.com, research@frederikweber.com

[†]these authors contributed equally to this work

‡these authors contributed equally to this work

Supplementary Figure S1

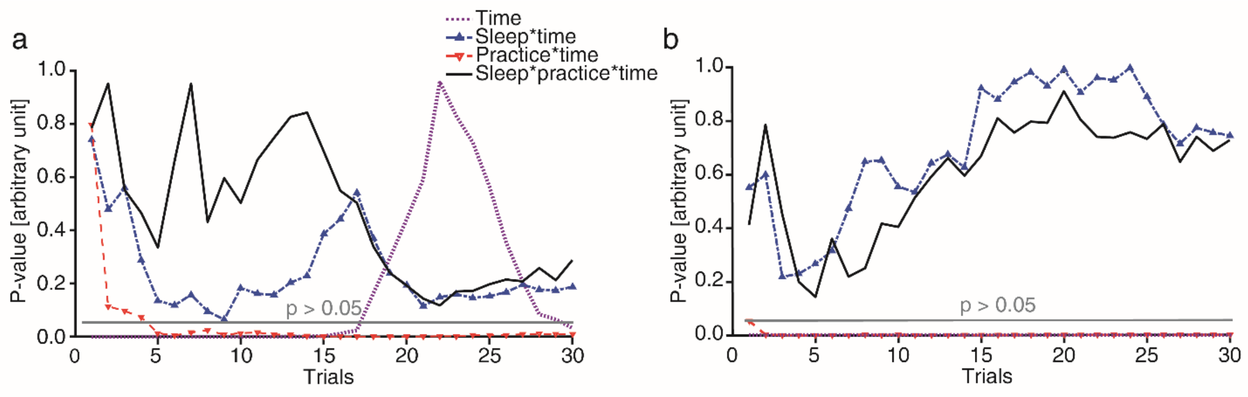

Figure S1: Explorative behavioral results. (**a**) Explorative analysis per trial, *p*-values of different factors and their interactions in mixed-model ANOVAs analyzing the consolidation effect from Training to Posttest; and (**b**) Training to Transfer. All p-values displayed in this figure were not corrected for multiple comparisons

Supplementary Table S2

ANOVA results of different behavioral parameters.

n = 48		AngleVp		PDmax		PDVmax		FeedbackPD	
		F	*p*	*F*	*p*	*F*	*p*	*F*	*p*
Adaptation	time	262.5	**<0.001**	133.6	**<0.001**	57.9	**<0.001**	56.9	**<0.001**
	time*sleep	0.6	0.457	1.1	0.301	0.8	0.388	0.1	0.711
	time*practice	<0.1	0.847	2.3	0.134	2.9	0.096	0.5	0.489
	time*practice*sleep	<0.1	0.94	0.1	0.73	<0.1	0.896	<0.1	0.881
	sleep	0.1	0.748	0.1	0.793	0.7	0.398	<0.1	0.96
	practice	1.2	0.274	1.3	0.261	3.9	0.054	0.1	0.73
	sleep*practice	0.1	0.74	1.5	0.232	0.3	0.861	0.4	0.509
Consolidation	time	15.4	**<0.001**	1.2	0.273	13.6	**<0.001**	0.2	0.644
	time*sleep	0.5	0.484	1.8	0.189	0.5	0.479	0.1	0.756
	time*practice	<0.1	0.897	4.6	**0.037**	10.0	**0.003**	0.1	0.739
	time*practice*sleep	<0.1	0.847	1.2	0.288	3.0	0.088	0.1	0.801
	sleep	0.3	0.568	<0.1	0.928	3.8	0.056	0.4	0.526
	practice	0.9	0.346	9.0	**0.004**	8.9	**0.005**	<0.1	0.951
	sleep*practice	<0.1	0.891	1.0	0.317	0.8	0.386	0.3	0.61
Generalization	time	618.9	**<0.001**	435.4	**<0.001**	351.2	**<0.001**	235.8	**<0.001**
	time*sleep	0.4	0.525	0.2	0.627	4.2	**0.045**	4.3	**0.044**
	time*practice	1.9	0.171	13.7	**<0.001**	19.8	**<0.001**	5.2	**0.028**
	time*practice*sleep	1.4	0.242	0.4	0.525	<0.1	0.849	0.9	0.361
	sleep	1.4	0.240	0.8	0.365	8.1	**0.007**	3.4	0.073
	practice	1.2	0.282	0.7	0.394	0.6	0.453	2.5	0.118
	sleep*practice	0.5	0.472	0.1	0.745	<0.1	0.938	0.3	0.569

This table shows uncorrected p-values of different ANOVAs performed for each research question and each performance parameter. Between subject factors are sleep (Wake, Sleep) and practice (Random,

Blocked). Within subject factor time changed according to the research question (adaptation: First Training Trials, Last Training Trials; consolidation: Last Training Trials, Posttest; generalization: Last Training Trials, Transfer). AngleVp describes the angle between a straight line and subject's trajectory at peak velocity. This parameter captures mostly feedforward mechanisms. PDmax and PDVmax capture the maximum perpendicular displacement (PD) of the trajectory and the PD at peak velocity. Both parameters are affected by feedforward and feedback mechanisms. FeedbackPD describes the difference from PD at peak velocity to 5% of the peak velocity at the end of the movement. This parameter reflects mostly feedback processes.

Supplementary Table S3

Correlations between EEG alpha power during training and consolidation

			ROI_left		ROI_right	
			Planning	Execution	Planning	Execution
Posttest	Blocked	*ρ*	0.3	0.22	0.33	0.23
		p	0.16	0.302	0.115	0.283
	Random	*ρ*	0.12	0.15	0.16	0.17
		p	0.57	0.486	0.47	0.412
Transfer	Blocked	*ρ*	0.6	0.55	0.39	0.49
		p	**0.003**	**0.006**	0.061	**0.017**
	Random	*ρ*	-0.1	0.04	0.08	0.2
		p	0.654	0.872	0.724	0.367

This table shows Spearman's rho [*ρ*] and uncorrected p-values of the associations between the EEG alpha power during training and the consolidation from Training-to-Posttest and Training-to-Transfer for the Blocked and Random groups.

Supplementary Table S4

Sleep states and correlations with motor adaptation and consolidation

Sleep parameter (n = 22)				Spearman rank correlations with behavior [ρ]				
Stages				Training	Posttest	Transfer	Posttest - Training	Transfer - Training
TST [min]	435.91	±	8.76	-0.219	-0.338	-0.152	0.160	-0.043
Sleep onset [min]	18.56	±	3.70	-0.092	-0.074	-0.011	0.040	0.179
WASO [%]	2.91	±	0.43	-0.238	-0.043	0.207	0.357	**0.433***
Stage 1 [%]	7.09	±	0.60	-0.342	-0.329	-0.152	0.123	0.040
Stage 2 [%]	45.23	±	1.52	0.234	0.342	-0.030	-0.174	-0.073
SWS [%]	27.64	±	1.50	0.043	-0.097	0.025	-0.019	-0.082
Non-REM [%]	72.86	±	0.84	0.420	0.281	0.058	-0.377	-0.172
REM [%]	17.14	±	0.71	-0.051	-0.095	-0.152	0.071	-0.158
Spindle power peak	13.32	±	0.11	**-0.500***	-0.163	0.072	0.367	0.363

Means ± s.e.m for the sleep parameters in the left columns. The right columns show Spearman's rank correlation of sleep parameters with behavioral measures of mean motor error (enclosed area) during end of Training, Posttest, Transfer, as well as changes from Training to Retest (Posttest – Training) and Training to Transfer (Transfer – Training). Note that for Posttest – Training and Transfer – Training a negative correlation is indicative of beneficial relation on consolidation success. Given are the total sleep time (TST), sleep onset (with reference to the time of lights off and beginning of first occurrence of stage 1-sleep epoch followed by stage 2-sleep), and time spent awake after sleep onset (WASO), sleep stage 1, sleep stage 2, SWS, non-REM (S2 + SWS) and REM in percentage of total sleep time. In addition, the sleep spindle power peak in the non-REM sleep power spectra of 12–15 Hz frequency range, Significant correlations are in bold, * $p < 0.05$, uncorrected for multiple comparisons.

Supplementary Table S5

Sleep parameters and events and correlations with motor adaptation and consolidation

Sleep parameter (*n* = 22)			Spearman rank correlations with behavior [ρ]				
Analysis	Property	Elec-trode	Training	Posttest	Transfer	Posttest - Training	Transfer - Training
Power density (Stage 2)	(0.5-4 Hz)	P3†	0.313	-0.152	0.017	**-0.501***	-0.252
Power density (REM)	(0.5-4 Hz)	C4†	0.339	-0.113	0.327	**-0.435***	-0.008
Spindle	duration	C3	**0.536***	-0.075	0.204	**-0.517***	-0.093
		C4†	**0.503***	-0.126	0.110	**-0.532***	-0.204
		Cz	**0.487***	-0.037	0.179	**-0.467***	-0.081
		F4‡	0.442	-0.251	0.150	**-0.453***	-0.093
		P3	**0.466***	-0.081	0.249	**-0.481***	-0.049
		P4	**0.430***	-0.125	0.277	**-0.517***	-0.010
		Pz‡	0.424	-0.183	0.284	**-0.549***	0.005
	frequency	C4†	**-0.535***	-0.101	0.109	**0.453***	**0.470***
		Cz	**-0.562****	-0.102	0.020	**0.470***	0.360
		Fz	**-0.573****	-0.141	0.054	**0.449***	0.411
Upstate spindle	chirp	C3	-0.348	-0.034	0.255	0.318	**0.430***
		C4†	-0.240	-0.018	0.330	0.384	**0.491***
		P3	-0.187	0.089	0.302	0.315	**0.450***
		P4	-0.223	0.072	0.266	0.273	**0.439***
	count	C3	0.266	-0.189	-0.316	**-0.482***	**-0.490***
		C4†	0.252	-0.187	-0.395	**-0.465***	**-0.547***
		Cz	0.248	-0.101	**-0.434***	-0.399	**-0.582****
		Fz	0.413	0.082	-0.268	**-0.456***	**-0.494***
		P3	0.382	-0.100	-0.379	**-0.548****	**-0.569****
		P4	0.233	-0.058	-0.397	-0.399	**-0.480***
		Pz‡	0.313	-0.122	-0.305	**-0.502***	**-0.454***
	density	C3	0.268	-0.153	-0.295	**-0.469***	**-0.481***
		C4†	0.310	-0.132	-0.319	**-0.486***	**-0.491***

	Cz	0.296	-0.146	-0.351	**-0.499***	**-0.548****
	F3‡	**0.498***	0.293	-0.226	**-0.451***	-0.441
	Fz	**0.426***	0.129	-0.258	**-0.436***	**-0.465***
	P3	0.343	-0.091	-0.368	**-0.518***	**-0.549****
	P4	0.311	-0.008	-0.318	**-0.462***	**-0.443***
	Pz‡	0.308	-0.123	-0.302	**-0.499***	-0.438
duration	C3	0.394	-0.198	-0.137	**-0.471***	-0.391
	C4†	**0.475***	-0.329	0.138	**-0.601****	-0.161
	Cz	**0.455***	-0.231	-0.013	**-0.615****	-0.295
	P3	0.369	-0.286	-0.047	**-0.505***	-0.252
	P4	**0.469***	-0.153	0.016	**-0.512***	-0.268
	Pz‡	0.353	-0.287	-0.205	**-0.544***	-0.439
frequency	C4†	**-0.492***	-0.071	0.081	**0.442***	**0.438***
	F3‡	**-0.498***	-0.265	0.171	0.367	**0.445***
	Fz	**-0.525***	-0.103	0.040	**0.436***	0.387
SD of slow-wave delay	Cz	-0.225	-0.196	0.302	0.125	**0.427***

Exploratory analysis of detailed sleep parameter's relation to consolidation during sleep. The right columns show Spearman's rank correlation of sleep parameters with behavioral measures of mean motor error (enclosed area) during end of Training, Posttest, Transfer, as well as changes from Training to Posttest (Posttest – Training) and Training to Transfer (Transfer – Training). Note that for Posttest – Training and Transfer – Training a negative correlation is indicative of a beneficial relation on consolidation success. Only relations are listed that reached the significant threshold for a relation with changes from either Training to Posttest or from Training to Transfer in any of the electrodes (Pz electrode location was excluded due to reduced sample size). Standard deviation (SD) of slow-wave delay was measured to the detected preceding slow wave down-state. All other measures did not reach significance for relations with these two consolidation measures. Some electrodes went bad or off in some subjects resulting in reduced number of subjects for correlations, † n = 21, ‡ n = 20. Significant correlations are in bold, ** $p < 0.01$, * $p < 0.05$, uncorrected for multiple comparisons.

Supplementary Methods

Example MATLAB code for computing the force field compensation factor running on MATLAB R2017a on Windows:

```
% x_forces: measured forces in x-direction
% y_forces: measured forces in y-direction
% velocityX: measured velocity in x-direction
% velocityY: measured velocity in y-direction

% compute trial direction vector (from start to target)
x_v1_raw = x_pos(1)-x_pos(end);
y_v1_raw = y_pos(1)-y_pos(end);
vec1_raw = [x_v1_raw; y_v1_raw];

% compute vector orthogonal to target direction
FFMatrix = [0 -1; 1 0]; % for CCW force field
vec2_raw = FFMatrix * vec1_raw;

% compute projected force in orthogonal direction
for v = 1:length(samples)
    vecForce_raw = [x_forces(v);y_forces(v)];
    proj_force(v) = norm((dot(vecForce_raw,vec2_raw) / dot(vec2_raw, vec2_raw)) *vec2_raw);
    VecAngle(v) = rad2deg(atan2(norm(cross([vecForce_raw' 0], …
   [vec2_raw' 0])),dot(vecForce_raw, vec2_raw)));
end
VecAngle = VecAngle > 90;
proj_force(VecAngle) = proj_force(VecAngle)*(-1);

% compute ideal force according to the trials velocity
velocity = [velocityX'; velocityY'];
FFMatrix = [0 15;-15 0]; % example force field matrix in CW direction
forceIdealArray = FFMatrix * velocity;
idealForce = [];
for fct=1:length(forceIdealArray)
    idealForce(fct)= sqrt(forceIdealArray(1, fct)^2 + ...
    forceIdealArray(2, fct)^2);
end

% compute fit with degree of 1 and compute the percentage force- field compensation
p = polyfit(idealForce, proj_force(1:end-1), 1);
FFC_factor = p(1)*100;
```
